# Supplementary material for: Neurodevelopmental Toxicity of Emamectin Benzoate to the Early Life Stage of Zebrafish Larvae (Danio rerio)
Source: Int J Mol Sci. 2023 Feb 13;24(4):3757. doi: 10.3390/ijms24043757 (PMC9964762; doi:10.3390/ijms24043757)
Supplement: Supplementary file 1 [file ijms-24-03757-s001.zip › ijms-2162740-supplementary.pdf]

Supplementary Table S1.

Supplementary Table S1. Sequences of primers for the genes tested.

Target Gene	GenBank Accession No.	Primer Sequences
β-actin	AF025305	Forward: 5’-ACAGGGAAAAGATGACACAGATCA-3’
		Reverse: 5’-CAGCCTGGATGGCAACGTA-3’
Cat	NM_130912.2	Forward: 5′-AGGGCAACTGGGATCTTACA-3′
		Reverse: 5′-TTTATGGGACCAGACCTTGG-3′
Sod	BX055516	Forward: 5′-GTCCGCACTTCAACCCTCA-3′
		Reverse: 5′-TCCTCATTGCCACCCTTCC-3′
Cu/Zn-Sod	NM_131294.1	Forward: 5′-GTCGTCTGGCTTGTGGAGTG-3′
		Reverse: 5′-TGTCAGCGGGCTAGTGCTT-3′
gat1	NM_001007362	Forward: 5’- ATGCTGTTTATCCTGTTCATCCG-3’
		Reverse: 5’- TGTTGAAGGGGTTGTAGCTCC-3’
gabra1	NM_001077326	Forward: 5’- TCAGGCAGAGCTGGAAGGAT-3’
		Reverse: 5’- TGCCGTTGTGGAAGAACGT-3’
gad1b	NM_194419	Forward: 5’- AACTCAGGCGATTGTTGCAT-3’
		Reverse: 5’- TGAGGACATTTCCAGCCTTC-3’
abat	NM_201498	Forward: 5’- GCGTTCAGGCAAAGCTCT-3’
		Reverse: 5’- GCAGGACGGAAACGGAT-3’
glsa	NM_001045044.1	Forward: 5’- AGGCCATGCTGAGGTTG -3’
		Reverse: 5’-CTGCCGTGCTGAGGTTG-3’
syn2a	NM_001002597	Forward: 5’-GTGACCATGCCAGCATTTC-3’
		Reverse: 5’-TGGTTCTCCACTTTCACCTT-3’
gfap	NM_131373	Forward: 5’- GGATGCAGCCAATCGTAAT-3’
		Reverse: 5’- TTCCAGGTCACAGGTCAG-3’
elavl3	NM_131449	Forward: 5’- AGACAAGATCACAGGCCAGAGCTT-3’
		Reverse: 5’- TGGTCTGCAGTTTGAGACCGTTGA-3’
shha	NM131063.3	Forward: 5’-TGTCCTCGACAACTCAACGG -3’
		Reverse: 5’- TCCGTGTATATCCGCTGCAC -3’
gap43	NM_131341	Forward: 5’- TGCTGCATCAGAAGAACTAA-3’
		Reverse: 5’- CCTCCGGTTTGATTCCATC-3’
Nrd	NM_130978.2	Forward: 5′- CCTCATTTGACGGACCCCTTAG -3′
		Reverse: 5′- AGACCCGCTGCCTGATAGTGC -3′
foxA3	NM_131299.1	Forward: 5’- TCTCAGCTTAGGCTGTTTGTAAGAT -3’
		Reverse: 5’-AAAAATACATCATGGCAATTTCATT-3’
pbx1a	AJ245962.1	Forward: 5’- GAGGGAAGAAAACAGGACATTG -3’
		Reverse: 5’-TTTCTTTTATTTCACACAGGACGTT-3’
mnx1	NM_001009885.2	Forward: 5’- AAACTCACAGCCAGCTCCTTT -3’
		Reverse: 5’-AAGCGTTTTGGTCTGGATAGATATT-3’
has2	NM_153650.2	Forward: 5’- GAAGGTTTGTAGAGTGTCTGGAGAG -3’
		Reverse: 5’-TTGTTACAAGCTTATACCCCATGAT-3’

elovl1a	NM_001005989.3	Forward: 5’-CCACGACTATCTCTTGAAAAGAAC -3’
		Reverse: 5’-CGACATCAAGAACTCATAAACAGTG-3’
